# A comparative study assessing the efficacy and safety of radiofrequency ablation versus surgical treatment for osteoid osteoma: retrospective analysis in a single institution

**DOI:** 10.1186/s13244-024-01656-1

**Published:** 2024-03-22

**Authors:** Jasminka Igrec, Maria Anna Smolle, Michael Meszarics, Theresa Marie Godschachner, Jakob Steiner, Mira Feichtinger, Emina Talakic, Rupert Horst Portugaller, Andreas Leithner, Michael Fuchsjäger, Iva Brcic

**Affiliations:** 1 Division of General Radiology, Department of Radiology, Medical University of Graz, Graz, Austria; 2 Department of Orthopaedics and Traumatology, Medical University of Graz, Graz, Austria; 3 Diagnostic and Research Institute of Pathology, Medical University of Graz, Graz, Austria; 4 Division of Neuroradiology, Vascular and Interventional Radiology, Department of Radiology, Medical University of Graz, Graz, Austria

**Keywords:** CT, MRI, Osteoid osteoma, Radiofrequency ablation

## Abstract

**Objective:**

We aim to evaluate the efficacy of CT-guided percutaneous radiofrequency ablation (RFA) and surgical treatment in osteoid osteoma (OO) treated at the Medical University of Graz.

**Materials and methods:**

In a single-institution study, we analysed data from January 2005 to January 2021 of patients with histological/radiological diagnosis of OO. CT and MRI scans were reviewed for typical findings. Means (with SD) and medians (with IQR) were reported for normally and non-normally distributed variables. Differences between groups were assessed using chi-squared tests and t-tests.

**Results:**

One hundred nineteen patients (mean age: 21.6 ± 10.9 years; 63.9% males) with confirmed OO were retrospectively evaluated. 73 and 43 patients underwent RFA and surgery, respectively. In three cases, RFA combined with surgery was performed. Pre-intervention, 103 patients (88.8%) had undergone CT, and 101 had an MRI (87.1%). The nidus was confirmed in 82.5% of cases with CTs (85/103) and 63.4% with MRIs (64/101). The majority of nidi were located cortically (*n* = 96; 82.8%), most frequently in the femur (38 patients, 33.3%) with a median size of 8.0 mm (IQR: 5.0–12.0 mm). Median symptom duration before treatment was 6.0 (IQR: 4.0–13.0) months. The complication rate was 12.1% (14/116; 15.1% RFA vs. 7.0% surgery; *p* = 0.196). In total, 11.2% of patients had persistent symptoms after one week with clinical success rates of RFA and surgery, 86.3% and 90.7% (*p* = 0.647), respectively.

**Conclusion:**

Compared to surgical treatment, CT-guided percutaneous RFA is a safe, minimally invasive, reliable, and efficient treatment option for OO.

**Critical relevance statement:**

This article critically assesses the diagnosis and treatment of osteoid osteoma, emphasising accurate imaging, and detailing a non-invasive option for effective management.

**Key points:**

• This study analyses 116 cases of OO at one institution, focusing on symptom persistence, recurrence in short-term follow-up, and complications in two study groups.

• Surgery showed higher, though not statistically significant, success despite comparable symptom persistence; CT displayed typical OO features more than MRI, regardless of the intramedullary, cortical and subperiosteal location as well as the site of the affected bone.

• CT-guided RFA is an effective therapeutic alternative for OO compared to surgical intervention. In case of atypical OO appearance, RFA is not the first-line treatment.

**Graphical Abstract:**

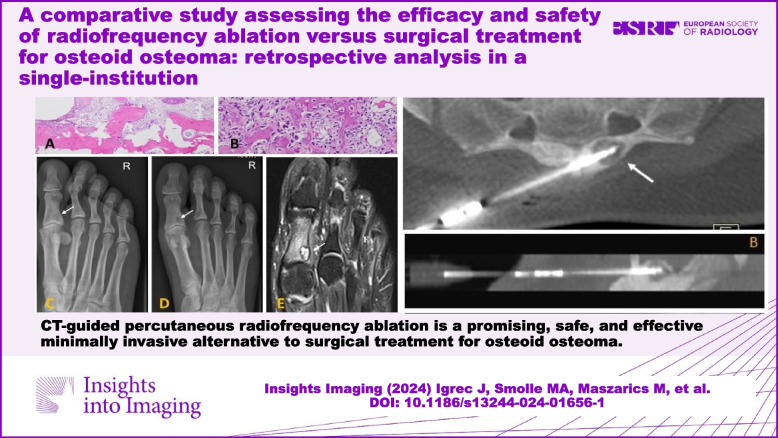

## Introduction

Osteoid osteoma (OO) is a benign bone tumour originating from osteoblasts, characterised by a tumour size typically not exceeding 2 cm. OO accounts for approximately 15% of cases among benign bone tumours, with the highest frequency observed during the second decade of life. The incidence of OO is more prevalent in male patients, and it predominantly affects the appendicular skeleton [1, 2]. 

OO typically presents as night pain relieved by salicylates [3, 4]. In active individuals, especially athletes, symptoms may mimic sport-related injuries like stress fractures, leading to delayed diagnosis [3].

**Fig. 1:**
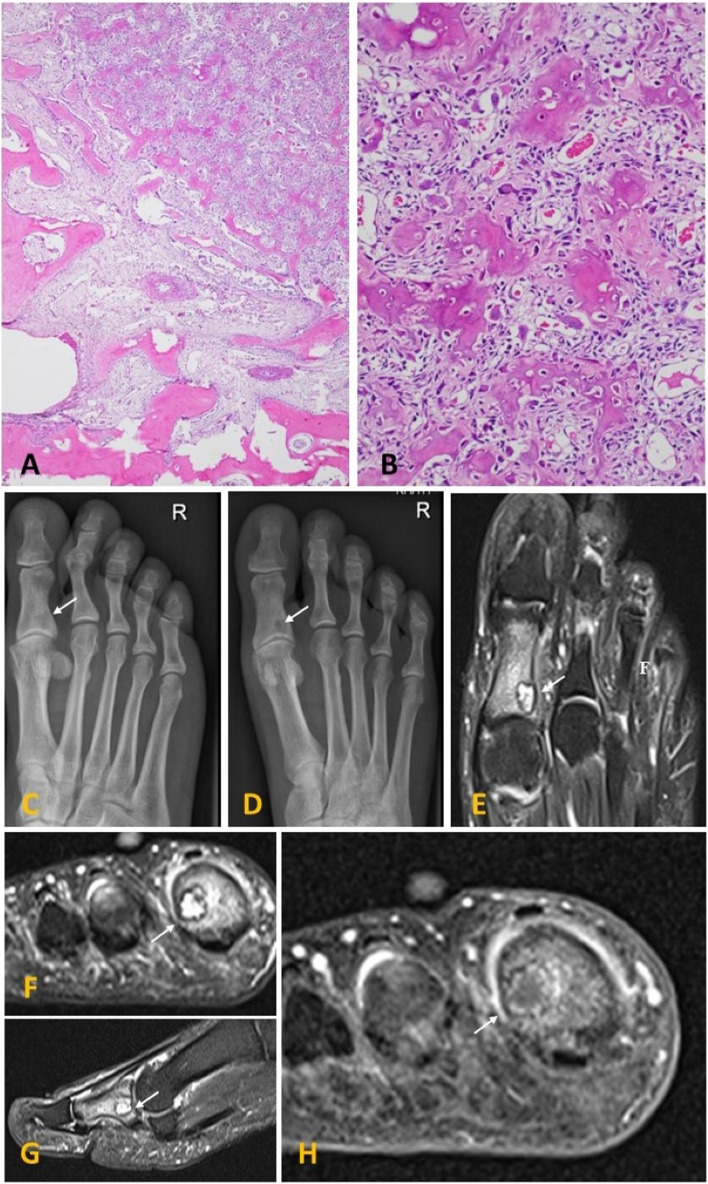
Osteoid osteoma of the base of the proximal phalanx of the first toe in a 55-year-old female patient. Histologically, the nidus is composed of a central area of vascularised fibrous tissue containing osteoblasts, surrounded by an area of sclerotic bone (**A**, **B**). Radiographs of the right foot in lateral (**C**) and AP (**D**) projections show a juxta-articular geographic osteolytic lesion with a thin sclerosed border with a lack of surrounding reactive sclerosis (arrow). Native and contrast MRI of the same foot (**E**–**H**). T2-weighted FS axial, coronal and sagittal MR images (**E**–**G**) depict oval hyperintense lesion with surrounding bone marrow oedema, periosteal reaction and oedema of surrounding soft tissues (arrow). Coronal T1-weighted FS image after intravenous application of gadolinium shows peripheral contrast enhancement of the lesion (arrow) and surrounding periost (**H**)

The OO can manifest intracortical, medullary, subperiosteally, or endosteally, with minor presentation differences based on location [2, 4–7]. OO is characterised by a centrally located nidus with a variable amount of calcification representing osteoid matrix and reactive changes of the surrounding bone, including cortical thickening, sclerosis (varying degrees, depending on location), and bone marrow oedema [8, 9]. The nidus has self-limiting growth, then usually becomes asymptomatic and spontaneously heals [10, 11]. Plain radiography is the initial imaging method of choice when evaluating nonspecific bone pain. However, its effectiveness in assessing lesions in anatomically challenging areas, such as the spine, pelvis, and hindfoot, is generally limited. To accurately detect the nidus and differentiate OO from other sclerotic bone lesions, the imaging modality of choice is computed tomography (CT). Optimal diagnostic accuracy is achieved by acquiring high-resolution thin-section axial and longitudinal multiplanar reformatted CT images, which are best evaluated using bone window settings. Magnetic resonance imaging (MRI) is considered inferior to CT in depicting and characterising OO, with a potential risk of up to 35% misdiagnosis [12]. However, MRI exhibits sensitivity in visualising bone and soft tissue oedema, osteitis, and synovitis, particularly in subarticular or intracapsular tumours [2, 13–16] (Fig. 1). Emerging techniques such as dynamic contrast-enhanced CT and MRI show promise in OO imaging. These methods allow for the visualisation of the more rapid early arterial enhancement of the nidus compared to avascular lesions [8, 11, 17–19].

RFA and surgery for OO depend on lesion specifics and location. Atypical lesions or those near critical structures, like nerves or blood vessels, may be less suited for RFA and may carry a higher risk of complications compared to minimally invasive surgical methods. Surgery offers direct access and is preferred for precise removal in complex cases. Treatment decisions are usually based on individual factors of the centre (like expertise in ablative techniques guiding); however, these should not solely determine the approach [11, 16, 20–22].

Our investigation aims to assess the rates of symptom persistence and complications during short-term follow-up in two distinct study groups.

## Materials and methods

### Patient selection

Ethical approval was obtained from the local Institutional Review Board (IRB) (EK 33–265 ex 20/21).

The patient selection for this study was conducted by retrieving data from a tumour database encompassing the period from January 2005 to January 2021. All patients with confirmed diagnoses and adequate clinical data were included in the study. Inclusion criteria were based on histological and/or radiological diagnosis confirming OO. CT and MRI were scrutinised to identify characteristic nidus formations. Exclusion criteria were (a) inadequate or unavailable CT or MRI images and (b) conservative treatment of patients with OO. Patients underwent RFA or surgery based on recommendations from joint discussions between radiologists and orthopaedic surgeons during the multidisciplinary clinical conference. Classification of complications was performed as suggested by Dindo et al. [23].

Follow-up involved clinical visits with collaborating orthopaedic surgeons 1-week post-treatment, marking the short-term follow-up phase. Clinical success was characterised by the alleviation of pain and restoration of normal function without the need for further treatment.

### Radiofrequency ablation

RFA was performed under general anaesthesia with a single dose of prophylactic intravenous 1 g cefuroxime. Following patient positioning and fixation, meticulous skin preparation and administration of local periosteal anaesthesia, precise preprocedural localisation of the nidus was achieved by acquiring contiguous CT scans on two different CT scanners. Until 2012, the CT scans were performed on the 4-slice CT scanner (Ge LightSpeed QX/I, GE Healthcare) using a reconstruction thickness of 2.5 mm. Since 2012, the CT scans have been performed on the 128-slice CT scanner (Somatom Definition AS + , Siemens Healthineers) using a reconstruction thickness of 2.4 mm. When in-plane access was not feasible, a stereotactic navigation system (CAS-One I ®; CAScination AG) was utilised to ensure accurate and precise localisation of the nidus. Under the guidance of CT, osseous access to the OO nidus was established using either a biopsy needle or a coaxial drill system. The choice of technique depended on the localisation of the nidus relative to the bone surface and the degree of adjacent bone sclerosis. Biopsy of the lesions was performed in cases where imaging results were inconclusive, aiming to exclude other potential pathologies. Following the biopsy, the biopsy needle was replaced by an active tip monopolar radiofrequency electrode of varying lengths (Rita StarBurst 14 G, AngioDynamics). CT imaging was conducted to verify the accurate placement of the electrode tip within the nidus. The electrode tip was then heated to a temperature of 90 °C for up to 6 min. This process created a spherical (for the 5-mm electrode) or a cylindrical ablation zone (for the 8-mm electrode), respectively. In cases involving larger tumours or off-centre electrode placement, the procedure was repeated with repositioned electrodes to ensure comprehensive coverage of the entire lesion. This approach aimed to induce coagulation necrosis within a defined area surrounding the electrode, encompassing all affected tissue.

Following the procedure, subsequent CT imaging was performed to confirm the absence of soft tissue swelling and hematoma. Depending on the localisation of the lesion, patients were advised to engage in partial weight-bearing activities for 2–3 weeks. In instances where post-interventional skin burns at the treated site occurred, likely secondary to the superficial location of the treated lesion, prophylactic antibiotic therapy was prescribed.

### Surgical technique

Patients underwent curettage of the lesion under regional, spinal, or general anaesthesia. The type of anaesthesia was at the discretion of the anaesthetist in charge. During the surgery, the skin and subcutaneous structures were meticulously prepared, followed by removing the lesion using curettes and high-speed burrs. First, a small cortical window was created for intramedullary lesions, while the procedure was performed directly on the bony cortex for cortical lesions. Depending on the lesion’s size, curettes of different angles and sizes were used and inter-exchanged during the procedure to reach all corners sufficiently. For high-speed burring (ANSPACH™ EG1™ High-Speed Electric System, DePuy Synthes, Warsaw, IN, US), burrs of varying size (between 3 and 9 mm, usually fluted ball shape) were chosen, again taking into consideration OO’s size and shape. The material obtained was sent for histopathological examination. Like RFA, patients were advised to partially weight-bear their affected extremities in case of large and lower-limb OOs for 2–3 weeks.

### Statistical analysis

For normally and non-normally distributed variables, means (with standard deviation (SD)) and medians (with interquartile ranges (IQR)) were provided. Differences between the two groups were compared with chi-squared tests for binary and categorical variables and *t*-tests for continuous variables. A *p*-value of < 0.05 was considered statistically significant. Stata Version 16.1 (StataCorp, College Station, TX, US) was used for statistical analysis.

## Results

### Demographics and baseline characteristics

One hundred nineteen patients (mean age at intervention: 21.6 ± 10.9 years; 63.9% (*n* = 76) males) with clinically and radiologically confirmed OO were retrospectively included. Most of the patients (73/119; 61.3%) underwent RFA, 43/119 (36.1%) surgery and three (2.5%) RFA combined with surgery. To allow for a comparison between patients treated with surgery or RFA alone, the three patients treated with both procedures were excluded from subsequent analyses, resulting in 116 cases being included.

Upon referral to our centre, 12.9% (15/116) of patients presented with relapse or persisting tumours. Six out of 15 patients had undergone RFA before referral, and the remaining 9 had surgery. In comparison, patients presenting with a primary tumour underwent RFA in 66.3% (67/101) and surgery in 33.7% (34/101) of cases, resulting in a statistically significant difference (*p* = 0.045). Patients´ demographics were summarised in Table 1.

**Table 1:** Patient demographics (*n* = 116) and differences between patients treated with RFA (*n* = 73) and surgery (*n* = 43)

	**Missing**	**Overall**	**RFA**	**Surgery**	***p*-value**
**Gender**					
Female	0	43 (37.1)	24 (32.9)	19 (44.2)	0.223
Male		73 (62.9)	49 (67.1)	24 (55.8)	
**Age** (mean ± SD; in years)	0	21.6 ± 11.0	18.8 ± 6.8	26.3 ± 14.7	** < 0.001**
**Symptom duration** (median (IQR); in months)	1	6 [4–13]	6 [3–12]	6 [4–24]	0.377
**Lesion size** (median (IQR); in mm)	0	8 [5–12]	7 [5–11]	8 [5–13]	0.441
**Location**					
Femur	0	38 (32.8)	30 (41.1)	8 (18.6)	**0.007**
Tibia		34 (29.3)	23 (31.5)	11 (25.6)	
Humerus		8 (6.9)	2 (2.7)	6 (13.9)	
Others		36 (31.0)	18 (24.7)	18 (41.9)	
**Localisation within bone**					
Cortical	0	96 (82.8)	63 (86.3)	33 (76.7)	0.431
Intracapsular		7 (6.0)	3 (4.1)	4 (9.3)	
Medullary		7 (6.0)	3 (4.1)	4 (9.3)	
Subperiosteal		6 (5.2)	4 (5.5)	2 (4.7)	
**CT scan**					
No	0	13 (11.2)	0 (0.0)	13 (30.2)	** < 0.001**
Yes		103 (88.8)	73 (100.0)	30 (69.8)	
**MRI scan**					
No	0	15 (12.9)	12 (16.4)	3 (7.0)	0.142
Yes		101 (87.1)	61 (83.6)	40 (93.0)	
**Procedure prior to referral**					
No	0	102 (87.9)	67 (91.8)	35 (81.4)	0.097
Yes		14 (12.1)	6 (8.2)	8 (18.6)	
**Complications**					
No	0	102 (87.9)	62 (84.9)	40 (93.0)	0.196
Yes		14 (12.1)	11 (15.1)	3 (7.0)	
**Outcome 1 week**					
Persisting symptoms	0	13 (11.2)	9 (12.3)	4 (9.3)	0.647
Declining pain		1 (0.9)	1 (1.4)	0 (0.0)	
Symptom free		102 (87.9)	63 (86.3)	39 (90.7)	

### Radiological, pathological, and functional outcomes

Before intervention at our centre, 103 patients (88.8%) underwent CT and 101 MRI (87.1%). The lesion was confirmed radiologically in 85/103 (82.5%) of CT scans and (64/101) 63.4% of MRIs. Median symptom duration before treatment amounted to 6.0 (IQR: 4.0–13.0) months. Definitive diagnosis was established at least six months after symptom onset in 57% of the patients.

The most frequent location of OO was the femur in 38 patients (33.3%), followed by the tibia in 34 (29.8%) and the humerus in 8 patients (7.0%). Most lesions were situated cortically (*n* = 96; 82.8%). OOs treated with RFA were significantly more commonly located in the femur (41.1%) or tibia (31.5%) compared to OOs undergoing surgery (18.6% femur, 25.6% tibia).

The median lesion size was 8.0 mm (IQR: 5.0–12.0 mm). In 64 cases, histopathological evaluation was performed, and in 59% (38/64) cases, the findings were consistent with OO. As expected, histopathological analysis was significantly more frequently available in patients treated with surgery (95.3%) than in the RFA group (31.3%; *p* < 0.001, Table 1).

The overall success rate in the RFA group was 86.3%, with postinterventional persisting symptoms in 12.3% of patients. In the surgery group, the success rate was 90.7%, with symptoms persisting in 9.3% of patients. The difference in overall success rate between the two groups was not significant (*p* = 0.647, Table 1).

### Complications

Complications were reported in 14 (12.1%) patients, three times more often in the RFA group, although these findings were not significant (*p* = 0.196, Table 1). In both groups, symptoms had regressed with equal frequency one week after intervention (*p* = 0.647). Three patients presented with nerve and three with thermal skin damage. Furthermore, one patient developed soft tissue inflammation resulting in a cutaneous fistula and one with a hypertrophic scar. Two patients experienced complications during anaesthesia; one had an anaphylactic shock, and the second developed an epiglottal swelling due to unknown drug allergies. During one procedure, a mechanical needle failure occurred, and the patient was further managed by removing the needle using a crown drill followed by RFA. Two patients developed joint contractures after the procedure, successfully treated by physiotherapy. Lastly, one patient with OO in tibial diaphysis experienced an insufficiency fracture (Table 2).

**Table 2:** Postoperative and postinterventional complications in the study cohort [23]

Grade	Type of complication	RFA (*n* = 11)	Surgery (*n* = 3)
1	Nerve damage	2	1
	Thermal skin injury	3	
	Soft tissue inflammation	1	
	Scar		1
	Joint contracture	1	1
2			
3	Insufficiency fracture	1	
	Mechanical needle failure	1	
4	Anaphylactic shock	1	
	Epiglottic swelling	1	

### Differences between RFA and surgery group

Patients undergoing RFA were significantly younger (*p* < 0.001) and more frequently underwent CT scans before intervention (*p* < 0.001) than those treated surgically (Table 1). Furthermore, lesions were more commonly confirmed on this imaging modality in patients later undergoing RFA (65/73 (89.0%) than those with surgery (20/30 (66.7%); *p* = 0.007). No difference regarding frequency (*p* = 0.142) or lesion confirmation at MRI (*p* = 0.158) was found between patients treated surgically (22/40 (55.0%)) or with RFA (42/61 (68.9%)).

Patients treated surgically more commonly underwent a procedure before referral to our centre than those later treated with RFA (*p* = 0.097; Table 1).

Complications occurred three times more often in patients treated with RFA than with surgery (*p* = 0.268), and pain had regressed within one week after intervention in both groups (*p* = 0.647).

## Discussion

**Fig. 2:**
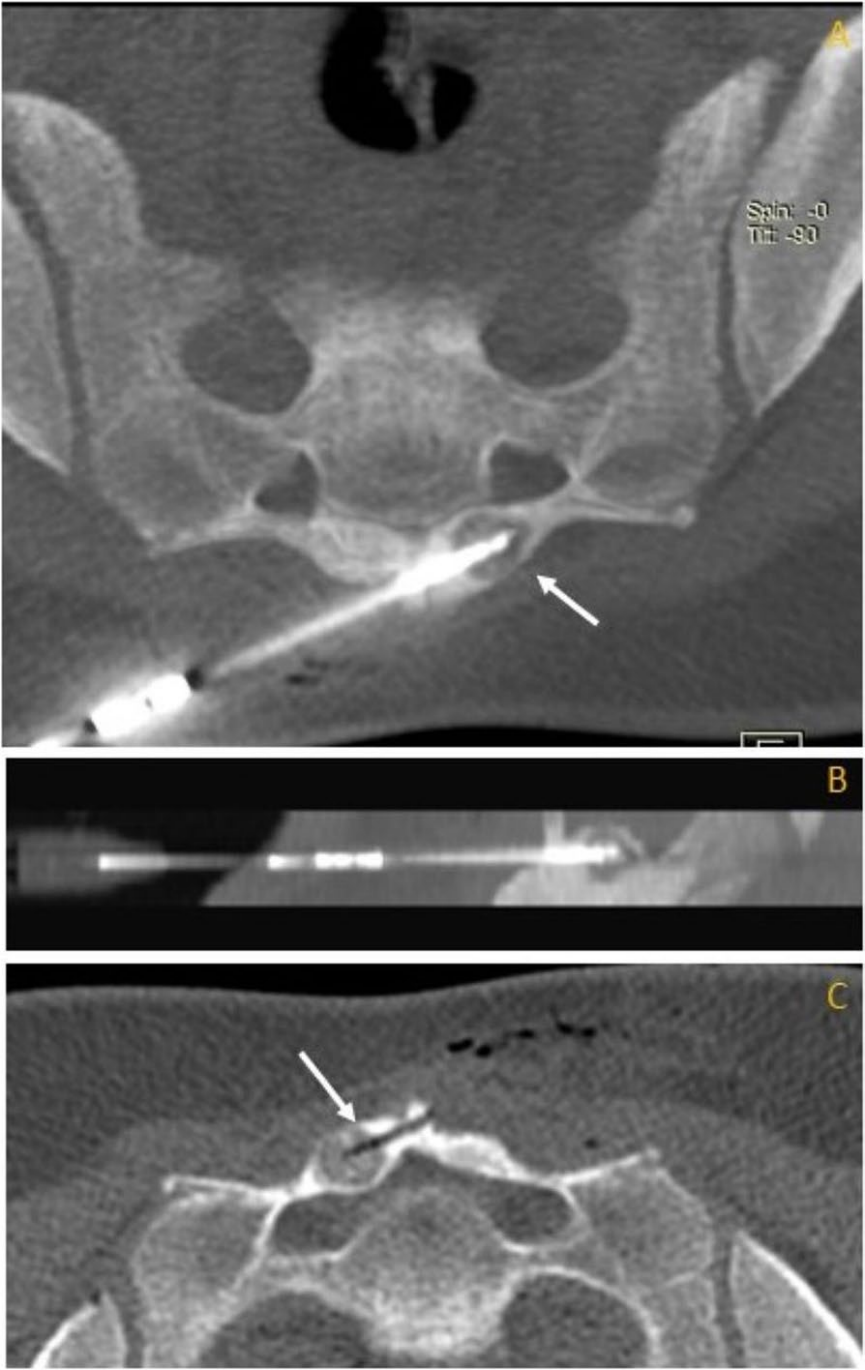
Radiofrequency ablation of an osteoid osteoma of the sacrum in a 16-year-old female patient. High-resolution CT of the sacrum in the bone window in axial (**A**) and sagittal (**B**) image during radiofrequency ablation depicts the intralesional placement of the needle tip inside the oval-shaped intracortical lying geographical osteolysis measuring CC 10 × LL 12 mm with pronounced perifocal sclerosis and no periosteal reaction, corresponding to osteoid osteoma (arrow). **C** Postprocedural bone defect and gas in the soft tissues on axial CT image after RFA (arrow). After 12 years of follow-up, the patient is recurrence-free

The primary goal of treatment for OO is the alleviation of pain. Since 1953, the treatment concept has changed significantly [1]. In 1992, Rosenthal described a novel method of placing radiofrequency electrodes in the centre of the nidus with complete relief of symptoms, revolutionising the treatment approach [22]. Nowadays, a CT-guided percutaneous RFA is regarded as a simple, minimally invasive, safe, and highly effective technique for treating OO, with reported primary success rates of up to 94% and secondary success rates between up to 100% [2, 24] (Fig. 2). Therefore, it is considered the modality of choice in most OO cases. Further development of minimal-invasive techniques enabled the treatment of even more challenging intra-articular and spinal lesions [24–29]. According to the literature, the success rate of percutaneous thermal ablation is high, with no significant difference between the efficacy and complication rates of various ablation methods [30].

**Fig. 3:**
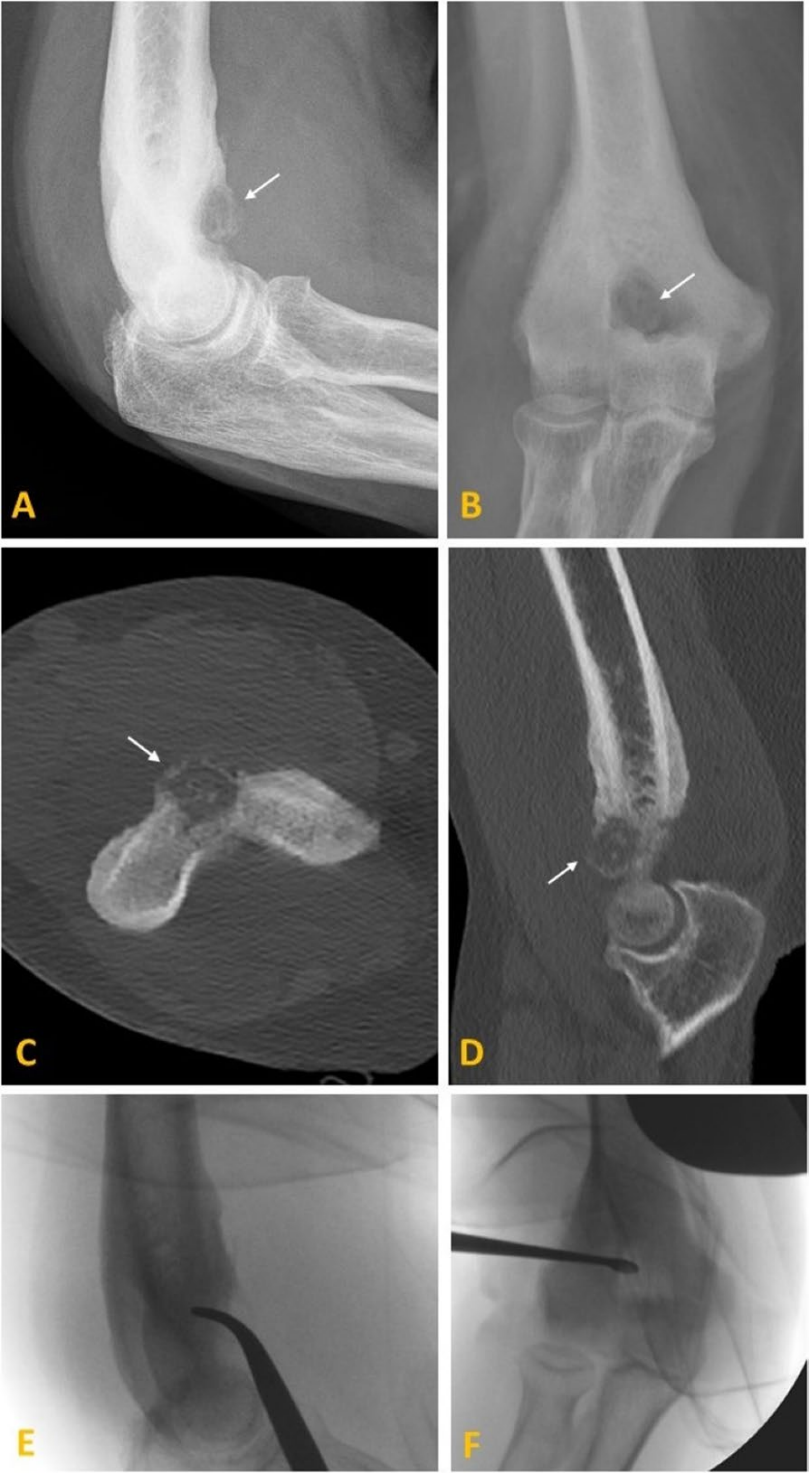
Curettage of an osteoid osteoma of the distal humerus in a 53-year-old female patient. Preoperative radiographs in lateral (**A**) and AP (**B**) projections show a juxta-articular geographic osteolytic lesion in the coronoid fossa (arrow) with surrounding reactive sclerosis and circumferential solid periosteal reaction. Corresponding high-resolution CT images in the bone window in axial (**C**) and sagittal (**D**) planes additionally show a thin neocortex formation and intralesional calcification. Intraoperative radiographs in lateral (**E**) and AP (**F**) projection during curettage

Surgical resection of OO is limited by the dissection size and, in rare cases, the need for cortical bone matrix transfer and internal fixation. Common surgical risks and complications include bleeding, infection, delayed wound healing, scar formation and risk for neurovascular damage. Complication rates reported after surgical treatment of OOs range between 9 and 28%, and post-resection pain persists in 7–20% due to recurrence [11]. Nevertheless, surgery still plays a significant role in patients with atypical imaging findings not responding to medical treatment or difficult anatomical locations (e.g. spine) in close vicinity to vital anatomical structures [22] (Fig. 3). In our study, we observed that the surgical treatment group demonstrated a higher overall success rate than the RFA group, 86.3% vs 90.7% of patients, respectively, with higher postinterventional symptom persistence, 12.3% vs 9.3% of patients, respectively. Moreover, it is noteworthy that RFA-treatment efficacy in our cohort was slightly lower than reported in the literature [11, 16, 21, 22]. This disparity can be attributed to two primary factors. Firstly, the limited availability of interventional radiology services in our institution might have influenced the outcomes. Secondly, patients admitted to our tertiary centre who were planned for surgical treatment had a higher likelihood of being previously treated at another institution, unlike patients undergoing RFA (*p* = 0.097; Table 1). Consequently, these organisational factors strongly influenced the results of our study, leading to surgical treatment being chosen for more than one-third of the cases within the cohort.

Although various studies have reported CT to be more sensitive and specific for evaluating OO compared to MRI, recent studies found that the combination of anatomical and functional sequences in multiparametric MRI, especially dynamic contrast-enhanced MRI and chemical-shift imaging, to be a helpful tool that significantly increases nidus conspicuity compared to nonenhanced MRI and CT [5, 18, 19, 31]. In the current study, most patients underwent both imaging methods during the diagnostic work-up, performed either at an external institution or our hospital, with typical imaging features present in 82.5% (85/103) of CT scans and 63.4% (64/101) of MRI scans. Additionally, there were no significant differences depending on tumour localisation (femur vs tibia vs humerus vs others) or their location within the bone (cortical, intracapsular, medullary, subperiosteal) regarding the presence of typical OO features on CT (*p*-value for localisation = 0.291; *p*-value for location within bone = 0.712) or MRI scan (*p*-value for localisation = 0.431; *p*-value for location within bone = 0.335). These results do not reflect the diagnostic algorithm in the work-up of suspected OOs at our institution, but rather highlight the risk of higher ionising radiation exposure of patients in whom OO was initially diagnosed with CT, and one of the percutaneous ablative techniques was considered a viable treatment option. This observation supports the worldwide trend of engaging multiparametric MRI as the preferred imaging method of choice before treatment and during follow-up of OOs, especially in paediatric patients [18, 24, 32–34].

It is frequently discussed whether the histological evaluation of the lesion diagnosed via imaging should be performed before treatment or whether the high specificity of cross-sectional imaging renders this additional procedure unnecessary [24, 30, 35]. Regarding the biopsy technique, surgical intervention procures a more substantial tissue sample for histopathological examination than the biopsy specimens obtained prior to CT-guided RFA. In the current study, tissue specimens were significantly more often available in the surgical group, and the diagnosis was more often confirmed in patients treated with surgery than in the RFA group (*p* < 0.001; Table 1). However, a biopsy was performed in half of the cases, and the diagnosis was confirmed histologically in 59% of cases. To rule out other pathologies, a biopsy should be performed in case of diagnostic uncertainty and atypical clinical presentation. In typical cases, pain relief after intervention within a short follow-up should be used as the main criterion of treatment success or failure [36, 37].

The main limitation of our study is its nonrandomised design, which inherently introduces selection bias due to its retrospective nature. To mitigate the impact of this limitation, we utilised a study population consisting of consecutive OO cases spanning over 15 years. Despite variations in sample sizes and surgeons between the RFA and surgery groups, the study maintained consistency in demographics and surgical approaches. However, an additional limitation arose from potential selection bias, as patients with unclear radiological findings and histology were excluded, possibly resulting in the underrepresentation of cases where imaging did not exhibit characteristic features, yet histological examination confirmed OO. Also, consideration should be given to the increasing establishment of RFA for OO during the observation period, potentially indicating the passage of a learning curve. An additional limitation is the lack of long-term follow-up data, which is crucial for assessing the recurrence and lasting effectiveness of OO treatments, providing a more comprehensive understanding of their long-term impacts. Finally, a prospective study with outcome analysis using a Visual Analysis Score would enable the assessment of the acute pain before intervention and during short-term follow-up, with this variable missing in the current series.

## Conclusion

Our study confirms that CT-guided percutaneous RFA is a simple, minimally invasive, safe, and highly effective optional alternative to surgery for treating OO with high clinical success rates in short-term follow-up. In case of atypical OO appearance, RFA is not the first-line treatment. Future research should investigate diverse biopsy techniques for diagnostic confirmation and assess the potential of multiparametric MRI as the primary imaging method, considering the risks of elevated ionising radiation exposure in CT-diagnosed cases. Despite its limitations, the study offers crucial insights into the changing landscape of OO treatment strategies, emphasising the necessity for prospective studies with comprehensive outcome analyses.

## Data Availability

Data is presented in the main paper.

## Abbreviations

CT: Computed tomography
IQR: Interquartile ranges
IRB: Institutional Review Board
MRI: Magnetic resonance imaging
OO: Osteoid osteoma
RFA: Radiofrequency ablation
SD: Standard deviation
